# Antagonists of Medical Authority

**Published:** 1913-09-06

**Authors:** 


### ANTAGONISTS OF MEDICAL AUTHORITY.

Many an institutional worker will remember well the scene that occurs when a case of smallpox, real or suspected, is found in the out-patient department. After it has been duly seen to and disposed of, the various hospital officers who have qualified themselves as "contacts" all go and get re-vaccinated. They go unquestioningly, being imbued with the spirit of obedience to the teachings of current medical science. The same course is probably recommended to, if not actually carried out upon, any patients who may also have been exposed to the risk of infection. But in this wider and more heterogeneous circle there may be, especially of late years, when "exemptions" have become so common, those who refuse the recommended prophylactic; and even in face of the utmost efforts of an energetic medical officer of health they could incur no serious disabilities by so doing. However, if the general circumstances and the conditions of time and of place be altered, such "conscientious objection" may entail a surprising degree of inconvenience. Of this fact a good instance was referred to in our Notes last week. Having accomplished the penultimate stage of their journey three politicians learn that, owing to smallpox at Sydney, unvaccinated persons will not be allowed to land. Three of them have not been vaccinated. Similarly embarrassed is a colonial Labour politician. However, more flexible apparently than Mr. Crooks at first, he bows himself in the house of Rimmon, takes the lymph, and is enabled to attend the parliamentary debate.

The details of these incidents are instructive. We have before enumerated in The Hospital those sorts and conditions of men, very various ones too, who are always found antagonistic to medical authority and medical privileges. There is the Dissenting minister who follows his teacher, the late Mr. W. T. Stead, in recommending a succession of cancer-curers, of faith-healers, of bone-setters, of phrenologists, fruitarians, and the like; and there are men of genius like Tolstoi, or of talent like Harold Frederic, strong anti-supernaturalists, who draw doctors as brutal, mercenary, and indelicate. There are our British Fabians, anti-vivisectionist and anti-vaccinationist almost to a man; and there are Labour leaders such as those now under notice, careless of culture, who encourage conscientious objection as much as any Hampstead intellectual. The common attitude of all these is explained by one or other of two nearly allied prepossessions—anti-authoritarianism and love of the weaker side. When the late "General" Booth lavished praise upon something known as blue electricity, vended by some one called Count Mattei, he was, as promoter of a junior religious organisation, in unconscious sympathy with the promoter of a junior therapeutic. The former was successful and the latter a failure, but that only shows the difference between religion and medicine. It was Tolstoi's revolutionary cast of mind which, going into detail, made him unable to forgive doctors their necessary privilege of seeing women half-naked. Mr. Shaw, too, in his smaller way, cannot stand the idea of one class of man being allowed to treat animals as it would be wrong for any other to treat them.

Our "reflection" (as Whistler would have said) is simply this: What a pity that the members of the crew of this galley have not the honour of each other's acquaintance! For they have not: they seem to be blind to each other's presence, ignorant of their solidarity. If they knew of that, there would be askance glances very soon; and in time a split. To point out and insist upon this strange fellowship is thus justifiable tactics to employ against our critics.

It may be left at that now; for the pro-vaccination case is much too long to be here argued. One piece of quite independent evidence might, however, be mentioned. Is, say, Mr. Crooks aware that a noted mathematician, a man of rationalistic views, with no medical axe to grind, and who as a fact is opposed to many medical measures, has pronounced from an examination of statistics that there is pretty close correspondence between the number of vaccination marks and the chances of escaping smallpox or of coming off lightly? Perhaps not. It all comes back, in fact, to Cromwell's invaluable advice: One should (nearly always) conceive it possible one may be mistaken; especially, it might be added, when the contingent penalty exists of wasting a twenty-thousand-mile journey.

